# Risikoidentifikation bei Polypharmazie in einer Pflegeheimpopulation

**DOI:** 10.1007/s00391-021-01850-6

**Published:** 2021-02-11

**Authors:** Michael Specka, Maria Groll, Norbert Scherbaum, Jens Wiltfang, Jens Benninghoff

**Affiliations:** 1 LVR-Klinikum Essen, Klinik für Psychiatrie und Psychotherapie, Universität Duisburg-Essen, Duisburg-Essen, Deutschland; 2 Klinik für Psychiatrie und Psychotherapie, Universitätsmedizin Göttingen, Göttingen, Deutschland; 3 Zentrum für Altersmedizin und Entwicklungsstörungen (ZfAE), kbo-Klinikum München-Ost, Vockestr. 72, 85540 Haar, Deutschland

**Keywords:** Polypharmazie, Multimorbidität, Medikamenteninteraktion, Medikamentennebenwirkungen, PRISCUS-Liste, Polypharmacy, Multimorbidity, Drug interaction, Drug side effects, PRISCUS list

## Abstract

**Einleitung:**

Multimorbidität im Alter ist u. a. ein Grund für intensivierte Pharmakotherapie. Gleichzeitig kann es mit steigender Medikamentenzahl zu einer Zunahme der Multimorbidität kommen, insbesondere wenn Interaktionen zwischen den Wirkstoffen zu unerwünschten Arzneiwirkungen (UAW) führen. Ziel dieser Untersuchung war es, in einer Pilotstudie Patienten zu identifizieren, die einem erhöhten Risiko für UAW unterliegen.

**Methoden:**

In einer Querschnittserhebung wurden 918 in Heimen lebende psychiatrisch behandelte Alterspatienten untersucht (Altersmittel: 79,3 (±11,6) Jahre; 31,8 % Männer). Es kamen exemplarisch verschiedene Methoden zur Identifikation von möglichen Risikopatienten zur Anwendung: eine die Interaktionen der Gesamtmedikation erfassende „Clinical-Decision-Support-Software“(CDSS)-Onlinedatenbank, mediQ, und eine Negativliste, die pauschal zu vermeidende Präparate indiziert, die PRISCUS-Liste.

**Ergebnis:**

Es hatten 76,3 % aller Studienpatienten bei Betrachtung der Gesamtmedikation ein klinisch relevantes Interaktionsrisiko (IR), 2,2 % standen unter einem darüber hinausgehenden potenziell starken UAW-Risiko durch Interaktionen. Ungefähr ein Viertel der untersuchten Studienpopulation erhielt potenziell inadäquate Medikamente gemäß PRISCUS.

**Schlussfolgerung:**

Diese unterschiedlichen Zahlen zeigen die Komplexität der eindeutigen Identifikation von Risikopatienten am Beispiel dieser beiden, auf unterschiedlicher Grundlage basierenden Instrumente. Trotz des technischen Fortschritts sollte der Schwerpunkt der UAW-Vermeidung unverändert darauf liegen, Medikamente erst nach besonders gründlicher Prüfung der klinischen Indikation zu verordnen und eine adäquate Verlaufskontrolle zu gewährleisten. Die neuen CDSS oder Negativlisten bieten hierbei Unterstützung.

## Abstract (en)

**Introduction:**

Multimorbidity in old age is one reason for intensified pharmacotherapy. At the same time, an increase in medications could augment multimorbidity, especially when drug interactions leading to undesired drug effects occur.

**Methods:**

In this cross-sectional study 918 mentally ill seniors living in nursing homes (mean age 79.3 (±11.6) years; 31.8% male) were included. Two different approaches to assess risks due to pharmacotherapy were applied: first mediQ, an online-based clinical decision support software (CDSS) and the PRISCUS list, which indicates potentially inappropriate medication. PRISCUS is the German equivalent to the American Geriatrics Society Beers criteria.

**Results:**

Of the patients in the study 76.3% were at clinical risk, 2.2% at potentially high risk for drug interactions regarding the entire medication as tested by mediQ, and about 25% of the studied population received potentially inappropriate medication according to the PRISCUS list.

**Conclusion:**

This difference clearly underlines the cumbersome complexity of identifying patients at risk by using these exemplary devices. The focus of avoiding undesired drug side effects should be taking medication only after thorough verification of clinical indications and under close monitoring. The CDSS or negative lists may support this process.

## Hintergrund und Fragestellung

Altersassoziierte Morbidität bedeutet häufig Multimedikation bzw. Polypharmazie. So nahm in Deutschland etwa ein Drittel der 18- bis 29-Jährigen ärztlich verordnete Medikamente ein, während es bei den über 65-Jährigen mehr als 85 % waren [6, 20]. Bei diesen Patienten kann u. U. schon der Funktionsabfall der Nieren oder Leber die Gefahr für unerwünschte Arzneimittelwirkungen (UAW) erhöhen.

UAW im Alter können zum vermehrten Auftreten von Krankenhausaufnahmen und im schlimmsten Fall zum Tod des Betroffenen führen [5, 17]. Um schwerwiegende Ereignisse dieser Art zu verringern, sollten Arzneiwirkstoffe mit hohem Risikopotenzial eher vermieden oder, wenn erforderlich, unter engmaschiger Beobachtung verabreicht werden.

In der vorliegenden Studie wurde das Risiko durch potenziell inadäquate Medikation (PIM) und Arzneimittelinteraktionen bei Bewohnern von Essener Alten- und Seniorenpflegeheimen untersucht, die durch die gerontopsychiatrische Ambulanz des LVR-Klinikums Essen/Kliniken und Institut der Universität Duisburg-Essen (im Folgenden LVR-Klinik Essen) betreut wurden. Das Arzneimittelinteraktionsrisiko wurde anhand einer „Clinical Decision Support Software – CDSS“, dem aus der Schweiz stammenden Programm mediQ (www.mediQ.ch), untersucht. Mit mediQ wurden sämtliche Präparate der Patienten auf ihr Interaktionsrisiko hin analysiert. Anhand der Ausprägung der festgestellten Interaktionsrisiken wurde insbesondere auf die Identifikation von Patienten mit hohem potenziellen Risikoprofil fokussiert. Ferner wurde untersucht, inwieweit die Patienten Medikamente erhielten, die in einer Liste für potenziell inadäquate Medikation (PIM), der PRISCUS-Liste [11], aufgeführt sind. Beide Methoden werden heute vielfach eingesetzt, um Risikopatienten für UAW zu identifizieren. Diese Studie sollte also neben der Identifikation von Risikopatienten Unterschiede zwischen den CDSS- und den PRISCUS-Risikogruppen untersuchen bzw. Überlappungen zwischen beiden Personenkreisen aufzeigen.

## Studiendesign und Methodik

### Stichprobe

**Figure 1:**
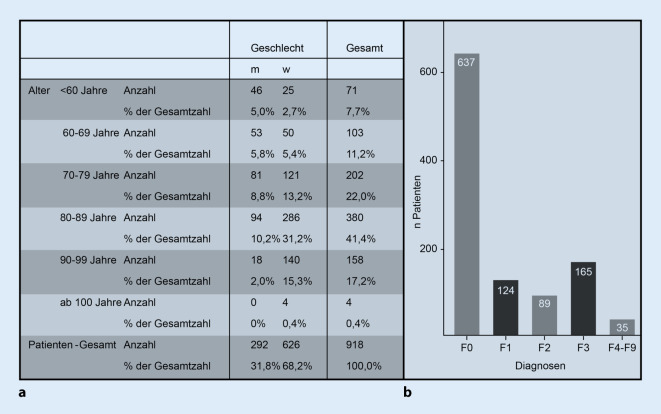

Die Untersuchungsstichprobe basierte auf einer Querschnittserhebung von insgesamt 918 Patienten mit einem mittleren Alter von 79,3 (±11,6) Jahren (weitere demografische Angaben: Abb. 1a). Diese Patienten waren sämtlich in Behandlung des gerontopsychiatrischen Zentrums des LVR-Klinikums Essen, Kliniken und Institut der Universität Duisburg-Essen. Die Behandlung erfolgte im Rahmen der psychiatrischen Institutsambulanz (PIA).

### Diagnose

Das Vorliegen einer schweren psychischen Störung, klassifiziert nach ICD-10, ist die Voraussetzung für die Aufnahme einer ambulanten Behandlung in einer psychiatrischen Institutsambulanz. Bei 637 (69,4 %) von insgesamt 918 Patienten wurde eine Störung aus dem Bereich organischer, einschließlich symptomatischer psychischer Störungen (F0) diagnostiziert, bei 124 (13,4 %) Patienten bestanden psychische und Verhaltensstörungen durch psychotrope Substanzen (F1), 89 (9,7 %) Patienten litten unter Schizophrenie, schizotypen oder wahnhaften Störungen (F2), 165 (18 %) Patienten hatten affektive Störungen (F3), 21 (2,3 %) zeigten sog. neurotische, Belastungs- und somatoforme Störungen (F4). Weiterhin bestanden folgende Störungsbilder: Verhaltensauffälligkeiten mit körperlichen Störungen und Faktoren (F5) bei 4 Patienten (0,4 %), Persönlichkeits- und Verhaltensstörungen (F6) bei 3 Patienten (0,3 %), Intelligenzminderung (F7) bei 6 Patienten (0,7 %), Verhaltens- und emotionale Störung mit Beginn in der Kindheit und Jugend (F9) bei einem Patienten (0,1 %). Psychiatrische Mehrfachdiagnosen wurden berücksichtigt (Abb. 1b).

### Untersuchung der Medikamentenwechselwirkungen

#### mediQ

Das mediQ-Programm des Qualitätszentrums für Medikamentensicherheit und Diagnostik der psychiatrischen Dienste Aargau AG (www.mediq.ch) diente zur Einschätzung des Interaktionspotenzials von medikamentösen Kombinationsbehandlungen. In mehreren Studien wurde die Software im stationären Behandlungssetting geprüft [8, 9]. Diese Arzneistoffinteraktionen wurden dann erneut mithilfe des Zurich Interaction System (ZHIAS) klassifiziert und in Interaktionsrisiken verschiedener Stärke graduiert. Dieses ZHIAS beinhaltet die Operational Classification of Drug Interactions (ORCA). Die Datenbank umfasst über 3000 Substanzen. Diese beinhalten neben Arzneimittelwirkstoffen auch Nahrungs- und Genussmittel. Ein Vorteil dieses Programms ist, dass das gesamte Spektrum der Arzneimittelwirkstoffe Berücksichtigung findet und es nicht auf Psychopharmaka (PPh) beschränkt bleibt. Die Informationsquellen zum Thema Interaktionen, auf die sich mediQ stützt, stammen aus Fachinformationen verschiedener Länder, so dem Arzneimittel-Kompendium der Schweiz, der Swissmedic Arzneimittelinformation sowie dem Fachinfoservice in Deutschland und dem Arzneispezialitätenregister aus Österreich sowie der European Medicines Agency (EMA) und der Drugs@FDA aus den Vereinigten Staaten. Daneben bezieht mediQ auch seine Informationen aus Onlinedatenbanken wie PubMed der US National Library of Medicine und vielen weiteren Quellen (www.mediq.ch). Bezüglich eines möglichen Interaktionspotenzials wurden die zu überprüfenden Arzneimittelwirkstoffe der Patienten in die Maske des Interaktionsprogramms mediQ eingegeben. Die Ergebnisse wurden jeweils in Form einer Kreuztabelle dargestellt. MediQ benutzt die Farbcodierung grau, gelb, orange und rot, um die verschiedenen Interaktionsstärken von 0 bis 3 zu veranschaulichen und rechnerisch damit umgehen zu können. Bei der Interaktionsstärke 0 (Farbe grau) sind keine relevanten, bei Interaktionsstärke 1 (Farbe gelb) in Ausnahmefällen relevante, bei Interaktionsstärke 2 (Farbe orange) klinisch relevante und bei der Interaktionsstärke 3 (Farbe rot) starke Interaktionen möglich (Tab. 1).

Die Analyse durch mediQ wurde sowohl für die psychopharmakologische (PPh) als auch für die Gesamtmedikation (PPh plus non-PPh) bei den jeweiligen Patienten durchgeführt.

#### PRISCUS-Liste

Ältere, multimorbide Menschen erhalten in der Regel eine Vielzahl an Arzneimitteln. Wegen eines erhöhten Risikos an unerwünschten Arzneimittelereignissen gelten bestimmte Arzneimittel bei älteren Patienten als potenziell inadäquate Medikation (PIM). Die deutsche Ausgabe der PIM-Liste wurde nach einer strukturierten Expertenbefragung (Delphi-Methode) erarbeitet: 83 Arzneistoffe aus 18 Arzneistoffklassen wurden als potenziell inadäquat für ältere Patienten bewertet. 46 Arzneistoffe konnten auch nach der zweiten Befragungsrunde nicht eindeutig eingestuft werden. Für den Fall, dass eine potenziell ungeeignete Medikation unvermeidbar ist, beinhaltet die endgültige PRISCUS-Liste Empfehlungen für die klinische Praxis wie beispielsweise Monitorparameter oder Dosisanpassungen. Ferner werden Therapiealternativen genannt [11].

**Table 1:** 

*mediQ* Medikamenteninteraktionsprogramm: Die Einschätzung des Interaktionspotenzials und möglichen Risikos wird farbcodiert grau, gelb, orange und rot in den verschiedenen für diese Studie adaptierten Interaktionsrisiken 0–3 dargestellt	
Interaktionsrisiko 0 – grau	Keine relevanten Interaktionsrisiken
Interaktionsrisiko 1 – gelb	In Ausnahmefällen relevant
Interaktionsrisiko 2 – orange	Mögliches, klinisch relevantes Risiko
Interaktionsrisiko 3 – rot	Hohes Risiko für potenzielle Interaktion

### Auswertung der Daten und Ethik

Die Daten wurden elektronisch mithilfe von SPSS (Pendler-Lizenz der Universität Duisburg-Essen) ausgewertet. Im Vorfeld wurde die Studie von der Ethikkommission der medizinischen Fakultät der Universität Duisburg-Essen (Aktenzeichen 11-4914-BO) geprüft und zugelassen.

## Ergebnisse

### Anzahl der verordneten Pharmaka

**Figure 2:**
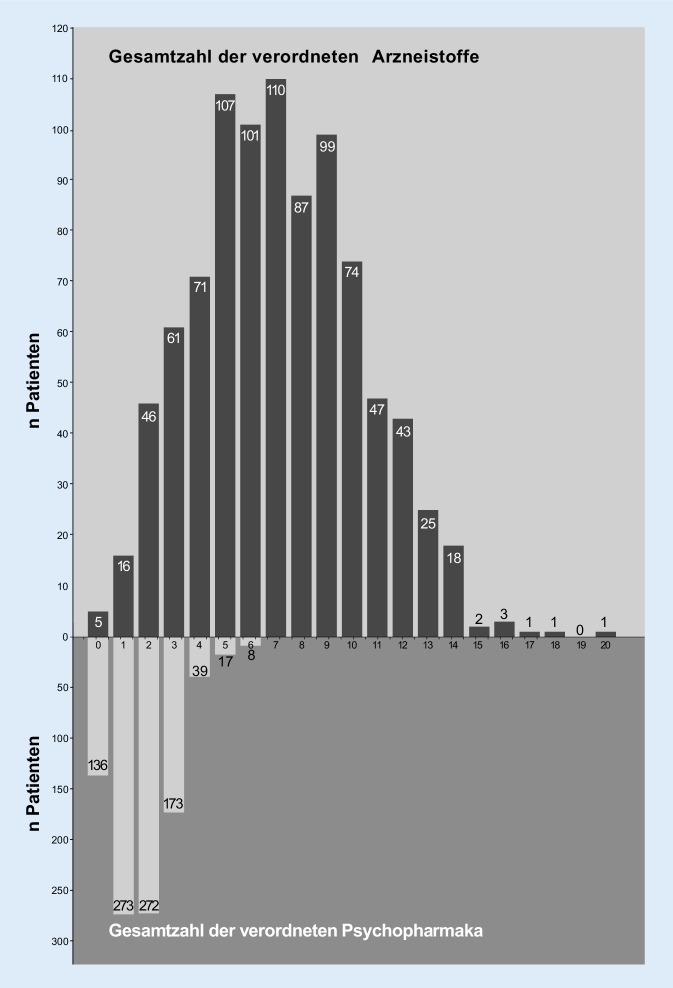

Die Verschreibungen sämtlicher Präparate lag im Median bei 7 (MW 7,1 ± 3,2), auf PPh bezogen lag der Median bei 2 (MW 1,8 ± 1,2). Die Spannweite reichte von 5 Patienten, die keine Verordnungen hatten, bis zu einem Patienten mit 20 verschriebenen Arzneistoffen (Abb. 2). Das Interaktionsrisiko (IR) durch 2 und mehr Substanzen in der Gesamtmedikation wurde bei den 897 Patienten (97,8 %), untersucht, die mindestens 2 Medikamente erhielten.

Insgesamt wurden 136 Patienten (14,8 %) ohne PPh behandelt. Zieht man von den verbleibenden 782 Personen die Patienten, die ein PPh einnahmen, ab (*n* = 273), konnten IR isoliert auf PPh bei 509 Patienten untersucht werden, was 55,4 % der Gesamtstichprobe entsprach.

### Analyse der Interaktionsrisiken mit mediQ und der PIM-Medikation anhand der PRISCUS-Liste

**Figure 3:**
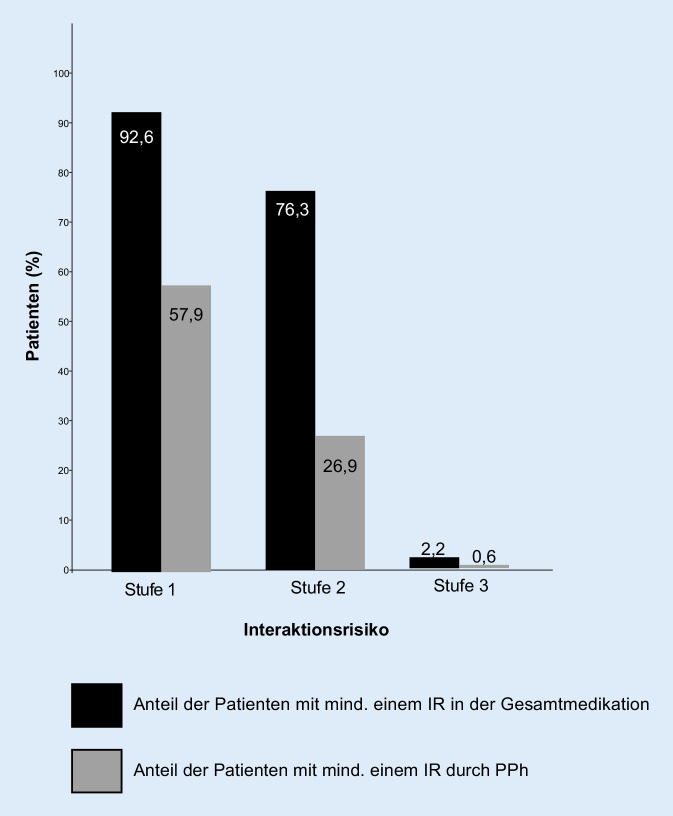

Die Auswertung mit mediQ ergab, dass 2,2 % aller Patienten (*n* = 20) das potenziell höchste IR (Stufe 3) aufwiesen. Bei der Charakterisierung dieser Patienten fiel auf, dass sie mit 8,8 (MW ±3,6 SD) mehr Wirkstoffverschreibungen als die Patienten der IR-Stufen 0, 1 und 2 mit 7,1 (MW ±3,2 SD) hatten. In der Stufe-3-Gruppe befanden sich 8 Männer und 12 Frauen. Diagnostisch waren demenzielle Erkrankungen (F0) mit 60 % führend, während sich die übrigen psychiatrischen Diagnosen in etwa gleich verteilten. Ferner ergab unsere Untersuchung IR der Stufe 2 bei 76,3 % der Patienten. IR der Stufe 1 waren bei 92,6 % von 918, also 850 Patienten, vorhanden (Abb. 3). Bei isolierter Betrachtung der ausschließlich PPh betreffenden IR zeigten sich – bezogen auf die Gesamtzahl von 782 mit PPh behandelten Patienten – bei 5 Personen (0,6 %) die IR-Stufe 3, bei 26,9 % (*n* = 210) die Stufe 2 und bei 57,9 % (*n* = 453) ein IR der Stufe 1.

Mit der PRISCUS-Liste für PIM ergaben sich folgende Ergebnisse: Insgesamt bekamen 24,5 %, d. h. 225 Patienten, Medikamente, die in dieser Liste als potenziell inadäquat aufgelistet sind. Bezogen auf PPh ergab sich, dass 133 Personen auf der PRISCUS-Liste als potenziell inadäquat eingestufte Substanzen erhielten (14,5 % der Gesamtstichprobe).

### Vergleich der eingesetzten Instrumente

**Figure 4:**
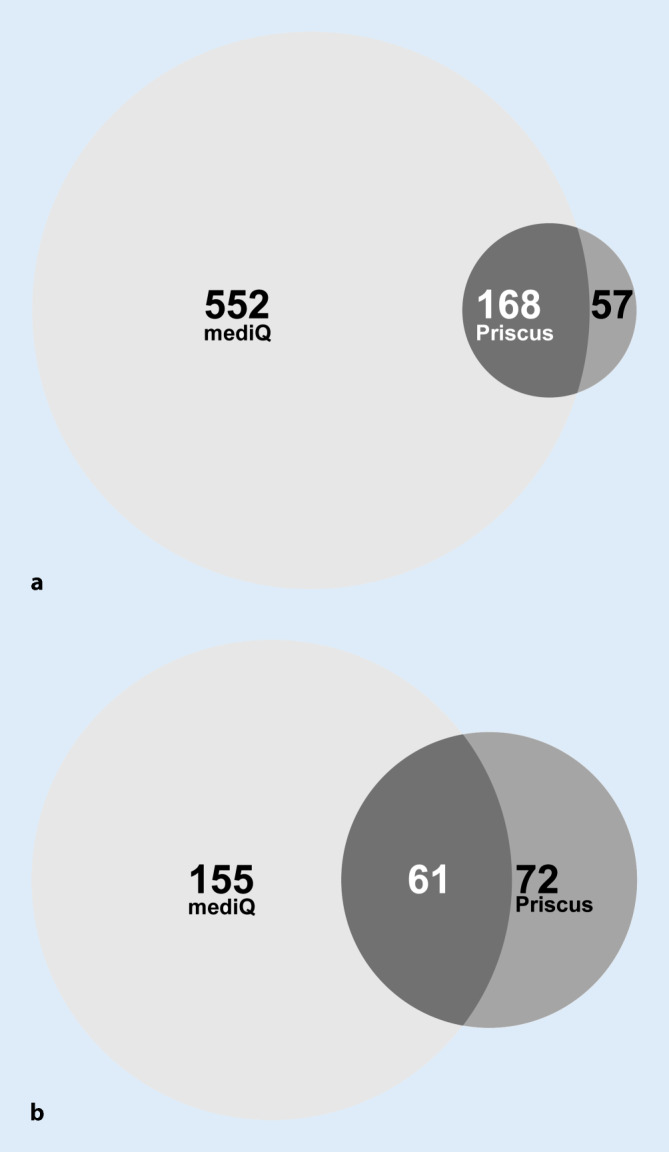

Gemäß PRISCUS-Liste erhielten 225 Patienten PIM. Davon waren 74,7 % (*n* = 168) laut mediQ in den IR-Stufen 2 und 3. Bezogen auf die Einnahme von PIM-PPh, die auf der PRISCUS-Liste geführt wurden, waren von den 133 betroffenen Patienten nach mediQ-Analyse 61 Patienten (45,9 %) im Bereich der IR-Stufe 2 oder 3 (Abb. 4).

## Diskussion

In dieser Studie haben wir pharmakologische IR bei fast 1000 Seniorenheimbewohnern in Essen mithilfe der CDSS mediQ untersucht; mediQ wurde wegen seiner im Vergleich zu weiteren Kandidaten guten Praktikabilität und breiten Datenbasis, wie auch ein Vergleich mit weiteren CDSS zeigte [8], ausgewählt. Daneben wurde mit der PRISCUS-Liste die Einnahme von potenziell inadäquater Medikation (PIM) erfasst.

Aus den hier erhobenen Daten lässt sich folgern, dass bei der untersuchten Population IR bei rund drei Viertel nach Auswertung mit dem CDSS mediQ potenzielle Risiken von klinischer Relevanz vorlagen, allerdings nur bei 2 % IR in der höchsten Stufe. Die Verordnung von PIM laut PRISCUS-Liste lag demgegenüber mit rund einem Viertel der Untersuchten niedriger. Bei abgetrennter Betrachtung der Psychopharmaka zeigte sich jeweils eine deutlich niedrigere Rate potenzieller IR bzw. in der Verschreibung von PIM (ein Viertel bzw. ein Siebentel der Patienten).

Hierbei ist zu betonen, dass es sich bei sämtlichen IR, deren Einteilung originär auf dem Zurich Interaction System (ZHIAS) und dem Operational Classification of Drug Interactions (ORCA) beruht, um potenzielle Risiken handelt. Dies bedeutet, dass die Ergebnisse der CDSS hinsichtlich ihrer klinischen Relevanz einer individuellen Überprüfung bedürfen.

Bei Bewohnern von Pflegeheimen besteht eine hohe Verschreibungsrate – auch im Hinblick auf PPh [3]. Zusammen genommen mit non-PPh erhielten Bewohner von Pflegeeinrichtungen im Durchschnitt 8,8 Präparate insgesamt [10]. In der vorliegenden Untersuchung bekamen die Patienten durchschnittlich 7,1 Medikamente rezeptiert. Die PPh-Medikation lag in unserem Kollektiv im Durchschnitt bei 1,8 Präparaten. Diese Ergebnisse sind vergleichbar mit einer kürzlich durchgeführten Untersuchung in brandenburgischen Pflegeheimen [14].

Neben der Nutzung von Negativlisten wie der PRISCUS-Liste gibt es ebenso den Ansatz der optimierenden Pharmakotherapie von älteren Menschen, der Medikamente in Bezug auf ihre Alltagstauglichkeit in einem Klassifikationssystem bewertet, z. B. die sog. FORTA-Liste („Fit for the aged“) [7]. Ferner sei angemerkt, dass trotz weiter Verbreitung von Polypharmazie Studien zeigten, dass bei bis zu 60 % und mehr der Patienten eine Unterversorgung für dringend indizierte Medikamente bestand [22]. Zu deren Identifizierung ist weiterhin die sog. START-Liste („screening tool to alert doctors to the right treatment“) zu nennen: Hierbei handelt es sich um eine Auflistung von evidenzbasierten Verordnungsindikationen und Behandlungsempfehlungen bei älteren Menschen [15]. Wir empfehlen zur Vertiefung der Thematik listenbasierter Ansätze in der Arzneitherapie eine in dieser Zeitschrift erschienene Übersichtsarbeit von Thiem [18].

Mögliche Begrenzungen der Studie bestanden u. a. darin, dass es sich um eine anonymisierte Datenauswertung handelte: So konnten wir weder die Patienten direkt identifizieren noch das mit der CDSS mediQ ermittelte Risikopotenzial auch tatsächlich beim Patienten verifizieren. Auch war es nicht möglich, sich direkt die Patienten herauszusuchen, die PIM entsprechend der PRISCUS-Liste erhielten. Nur mithilfe der individuellen Patientenakte wäre es dann mit Labor, Vorbefunden etc. möglich gewesen, den abstrakt analysierten potenziellen Risiken durch Maßnahmen zu begegnen bzw. den jeweiligen Patienten strenger zu beobachten und die Indikationen noch genauer zu hinterfragen.

Schließlich lässt sich bei ambulanten Patienten noch schwerer die Gesamtmedikation erfassen, da zumindest nicht ausgeschlossen werden konnte, dass „Over-the-counter“(OTC)-Präparate und Bedarfsmedikation der Pflegeeinrichtungen nicht dokumentiert wurden.

Allgemeine Maßnahmen zur Optimierung von Medikamentenverordnungen für ältere Patienten in Senioren- und Pflegeheimen wurden kürzlich auf Basis eines systematischen Cochrane-Reviews von 12 Studien aus 10 Ländern diskutiert [19], wobei die Überprüfung der Medikation bei nur 10 Studien der für den Cochrane-Prozess ausgewählten Arbeiten erfolgte [1]. Das Evidenzniveau war bei „Medikationsreviews“ niedrig. Unerwünschte Folgen für die Patienten konnten nicht signifikant verringert werden. Auch zeigt eine jüngere, zunächst vielversprechende, clusterrandomisierte Studie aus den Niederlanden trotz aufwendiger Interventionen, u. a. unter Einbeziehung von Apothekern, in den definierten Endpunkten wie Stürzen, Häufigkeit von Visiten, neuropsychiatrischen Symptomen und „quality of life“ keine signifikanten Unterschiede auf [21]. Ähnlich uneinheitlich im Outcome blieb die COME-ON-Arbeit aus Belgien, die zwar eine PIM-Reduktion in der Interventionsgruppe zeigte – allerdings bei erhöhter Mortalität im Vergleich zur Kontrollgruppe [2]. Zu einem ähnlichen Resultat kam eine norwegische Interventionsstudie, in der zwar eine Reduktion in der Anzahl verordneter Medikamente erreicht werden konnte, diese aber mit reduzierter Lebensqualität in der Interventionsgruppe verbunden war [12]. Im Hinblick auf konkrete Risikokonstellationen sei auf detaillierte Arbeiten in dieser Zeitschrift verwiesen [13, 16].

Wir haben diese Studie durchgeführt, um das Ausmaß potenzieller Gefahren durch Polymedikation in der besonders vulnerablen Gruppe der multimorbiden Patienten zu erfassen und zu identifizieren. Identifizieren ließen sich bei fast 1000 untersuchten Patienten 2,2 %, die ein potenzielles Risiko einer starken Interaktion aufwiesen. Allein durch Psychopharmaka verursacht, besteht dieses Risiko bei 0,6 % der hier analysierten Patienten, darauf sei auch bei der gesellschaftlichen Diskussion zu diesem manchmal emotional aufgeladenen Thema hingewiesen. Diese potenziellen Risiken durch Interaktionen sind durch den jeweiligen Arzt immer ernst zu nehmen, und es ist unter strengen Bedingungen das Risiko von therapeutischem Nutzen und möglichem Schaden stets abzuwägen. Die untersuchten Hilfsmittel CDSS oder eine Negativliste können hier sicherlich einen Beitrag leisten, alleine reichen sie zur Identifizierung von Risikopatienten, wie die vorliegende Arbeit gezeigt hat, nicht aus – dafür sind die Ergebnisse zu heterogen. Es sei aber schließlich darauf hingewiesen, dass selbst monotherapeutische Ansätze durchaus ein erhebliches Gefahrenpotenzial für Ältere darstellen können; erwähnt sei hier nur die langfristige Verschreibung von hochpotenten Neuroleptika in hoher Dosis bei Menschen mit Demenz [4].

## Fazit für die Praxis

Aus der Gesamtschau der Studie ergeben sich aus Sicht der Autoren folgende Konsequenzen für Klinik und Praxis insbesondere in der Behandlung gerontopsychiatrischer Patienten:

1. Bei der Untersuchung des Interaktionspotenzials ist jeweils die Gesamtmedikation zu untersuchen; Einzelbetrachtungen nur auf Psychopharmaka bezogen sind eindeutig nicht hinreichend.
2. Es empfiehlt sich dringend, aufgrund der nicht mehr überschaubaren Interaktionen eine CDSS hinzuzuziehen.
3. Die Einordnung des tatsächlichen Risikos für den Patienten muss individuell und stets unter Abwägung von Medikamentennutzen und potenzieller Gefährdung erfolgen.
